# Supplementary material for: Theory Designed Strategies to Support Implementation of Genomics in Nephrology
Source: Genes (Basel). 2022 Oct 21;13(10):1919. doi: 10.3390/genes13101919 (PMC9601730; doi:10.3390/genes13101919)
Supplement: Supplementary file 1 [file genes-13-01919-s001.zip › genes-1941986-supplementary (3).pdf]

Supplementary material

Contents

File S1: Semi-structured Interview Schedule: Health Professionals 2
File S2: Barriers and Interventions Reference List 4
Table S1: CFIR coding table applied to Nephrology 6
Table S2: Spreadsheet for CFIR Coding analysis 14

File S1. Semi-structured Interview Schedule: Health Professionals

Thank you for agreeing to participate in this study. We have provided you with an information statement that describes the study, do you have any questions about your participation or the study in general? *If yes, answer questions. If no, proceed*

This interview will be conversational in style and I have some broad questions and prompts to guide us. If at any stage you do not wish to answer a question, please let me know and we can move on to another topic.

Do you give your consent to participate in this study as described in the participant information statement? *If yes, proceed with interview. If no, cease interview.*

Aim: to further explore barriers to the uptake of genomics by nephrologists based on the survey and recent studies, and to identify and prioritise relevant intervention strategies to facilitate the widespread implementation of genomics in nephrology

**Broad topic**	**Sample Questions**	**Prompts/additional information**
Introduction	What is your title and background?	• Organisation<br>• Qualifications<br>• Years' practice<br>• Did you participate in the survey y/n
	Describe your current role<br><br>Context	• What<br>• Private or public %<br>• Subspecialty (transplant, GN, dialysis etc)<br>• Do you have any experience with genomics? (eg RGC?)<br>• How does your department feel about genomics? (prompt – is it promoted or discouraged?)<br>• What is your hospital executive committee's stance on genomics<br>• Are these providers collaborating with genetics? Who is facilitating? Are patients being referred? Including additional analyses here can allow for a bigger picture of result disclosure amongst nephrologists.
Barriers	From survey responses and the literature to date, here is a list of some barriers, could you tell me your thoughts on this? Have we missed any?<br><br>What do you think are the top 3 challenges for nephrologists?	• please refer to list<br>• Maybe probe unusual ones – can you tell me why you think x is a challenge?
Interventions	Given the barriers we discussed earlier, what are	• Give some examples

	the top three interventions that are both useful and feasible?	
Preferences for model of service delivery (as an intervention)	From the survey there are three common preferences on how genomics services can be integrated: (list)<br>Have we missed any?<br>Right now, what would be your preferred model be?<br>How does this model help address the current challenges?<br><br>(prompt if time permits)<br>Is there any more that could be done to improve this model?<br><br>Do you see this changing over time?	• Nephrologist refers to multidisciplinary renal genetics' clinic<br>• Nephrologist orders test and returns result with clinical genetics support as needed<br>• Nephrologist refers to clinical genetics
Views on current and future practice	Do you have any further thoughts on this topic?	

File S2. Barriers and Interventions Reference List

BARRIERS

1. Organizational barriers (inner setting)

- Testing process is too difficult (eg ordering, counselling etc)
- Access to clinical genetics expertise
- Access to genetic clinics (is there a service, is it timely?)
- Access to genetics counsellor
- Hospital funding
- Prioritisation of genomics in the department/hospital
- Lack of genomics champion
- Support from unit/organisation to innovate change
- Not enough opportunity to learn about genomics

2. Higher level barriers (outer setting)

- No government reimbursement for testing
- No government support for review of patients with genetic conditions
- Genomics is not prioritized in nephrology community (ANZSN, funding grants, curriculum, etc)
- Comparable units are not implementing genomics

3. Clinician related barriers

- Lack of clinician confidence to use genomic testing

4. Specific barriers associated with genomics itself

- Genomics is too complex to use in nephrology
- Not clinically useful
- Not cost effective

5. Patient level barriers

- Patients are not requesting or interested in testing
- Patients cannot access testing

INTERVENTIONS

- Nominate a genomics champion for the unit (nephrology) to provide advice and improve links between clinical genetics and nephrology
- Easier access to local genetics expertise (whether it be a Genetics Counsellor, Clinical Geneticist, Renal Genetics Clinic)- improve the relationship
- Incorporate genomics into the ANZSN scientific update course and kidney school
- Offer nephrology trainees a 6-month rotation in clinical genetics versus dedicated 1yr Renal Genetics Fellowship positions

- Funding support for test and consultation reimbursement (both at a hospital and federal level)
- “Help Desk” services at diagnostic service providers
- State/Regional/Hospital virtual MDT case conferences
- Genomics Competencies in RACP nephrology curriculum

PREFERRED MODEL OF SERVICE DELIVERY

- Nephrologist refers to multidisciplinary renal genetics’ clinic
- Nephrologist orders test and returns result with clinical genetics support as needed
- Nephrologist refers to clinical genetics

Table S1: CFIR coding table applied to Nephrology

Here we have applied the theoretical domains from the CFIR to the context of nephrology to guide the deductive coding of the interviews.

Construct		Short Description	Description in nephrology context
**I. INTERVENTION CHARACTERISTICS**			
A	Intervention Source	Perception of key stakeholders about whether the intervention is externally or internally developed.	Perceptions of key stakeholders about whether the implementation of genomics in nephrology is externally or internally developed.
B	Evidence Strength & Quality	Stakeholders' perceptions of the quality and validity of evidence supporting the belief that the intervention will have desired outcomes.	Stakeholders' perceptions of the quality and validity of evidence supporting the belief that the use of implementation of genomics in nephrology will have desired outcomes
C	Relative Advantage	Stakeholders' perception of the advantage of implementing the intervention versus an alternative solution.	Stakeholders' perception of the advantage of implementing genomics in nephrology versus an alternative solution.
D	Adaptability	The degree to which an intervention can be adapted, tailored, refined, or reinvented to meet local needs.	The degree to which implementation of genomics in nephrology can be adapted,

			tailored, refined, or reinvented to meet clinical, lab or patient needs.
E	Trialability	The ability to test the intervention on a small scale in the organization, and to be able to reverse course (undo implementation) if warranted.	The ability to try out implementation of genomics in nephrology and to be able to reverse course (undo implementation) if warranted.
F	Complexity	Perceived difficulty of implementation, reflected by duration, scope, radicalness, disruptiveness, centrality, and intricacy and number of steps required to implement.	Perceived difficulty of implementing genomics in nephrology, including duration, scope, radicalness, disruptiveness, centrality, and intricacy and number of steps required.
G	Design Quality & Packaging	Perceived excellence in how the intervention is bundled, presented, and assembled.	Perception of how implementation of genomics in nephrology is presented, and assembled.
H	Cost	Costs of the intervention and costs associated with implementing the intervention including investment, supply, and opportunity costs.	Costs of the implementation of genomics in nephrology.<br>Plus associated costs e.g. investment, supply, and opportunity costs.
**II. OUTER SETTING**			

A	Patient Needs & Resources	The extent to which patient needs, as well as barriers and facilitators to meet those needs, are accurately known and prioritized by the organization.	The extent to which patient and family needs (inc barriers and facilitators to meet those needs), are accurately known and prioritised by the organization.
B	Cosmopolitanism	The degree to which an organization is networked with other external organizations.	The degree to which organisations are networked with other external organisations.
C	Peer Pressure	Mimetic or competitive pressure to implement an intervention; typically because most or other key peer or competing organizations have already implemented or are in a bid for a competitive edge.	Mimetic or competitive pressure to implement genomics in nephrology; *typically, but not always,*because most or other key peer or competing organizations have already implemented or are in a bid for a competitive edge.
D	External Policy & Incentives	A broad construct that includes external strategies to spread interventions, including policy and regulations (governmental or other central entity), external mandates, recommendations and guidelines, pay-for-performance, collaboratives, and public or benchmark reporting.	A broad construct that includes external strategies to spread interventions, including policy and regulations (governmental or other central entity), external mandates, recommendations and guidelines, pay-for-performance, collaboratives, and

			public or benchmark reporting.
**III. INNER SETTING**			
A	Structural Characteristics	The social architecture, age, maturity, and size of an organization.	The social architecture, age, maturity, and size of an organisation.
B	Networks & Communications	The nature and quality of webs of social networks and the nature and quality of formal and informal communications within an organization.	Nature and quality of i) social networks and ii) formal and informal communications within either the home organisation or the nephrology and genetics teams
C	Culture	Norms, values, and basic assumptions of a given organization.	Norms, values, and basic assumptions of either the home organisation or the nephrology and genetics teams
D	Implementation Climate	The absorptive capacity for change, shared receptivity of involved individuals to an intervention, and the extent to which use of that intervention will be rewarded, supported, and expected within their organization.	The absorptive capacity for change, shared receptivity of involved individuals to genomics in nephrology, and the extent to which use of genomics in nephrology will be rewarded, supported, and/or expected within their organisation.
1	*Tension for Change*	The degree to which stakeholders perceive the current situation as intolerable or needing change.	The degree to which stakeholders perceive the lack of genomics in

			nephrology as intolerable or needing change.
2	*Compatibility*	The degree of tangible fit between meaning and values attached to the intervention by involved individuals, how those align with individuals' own norms, values, and perceived risks and needs, and how the intervention fits with existing workflows and systems.	Values attached to genomics in nephrology aligned with personal norms and values, perceived risks and needs, and how genomics in nephrology fits with current ways of working and systems
3	*Relative Priority*	Individuals' shared perception of the importance of the implementation within the organization.	Individuals' shared perception of the importance of the implementation of genomics in nephrology in the organization.
4	*Organizational Incentives & Rewards*	Extrinsic incentives such as goal-sharing awards, performance reviews, promotions, and raises in salary, and less tangible incentives such as increased stature or respect.	Extrinsic incentives e.g. awards, performance reviews and less tangible incentives e.g. respect,
5	*Goals and Feedback*	The degree to which goals are clearly communicated, acted upon, and fed back to staff, and alignment of that feedback with goals.	The degree to which goals are clearly communicated, acted upon, and fed back to staff, and alignment of that feedback with goals.
6	*Learning Climate*	A climate in which: a) leaders express their own fallibility and need for team members' assistance and input; b) team members feel that they are essential, valued, and knowledgeable partners in the change process; c) individuals feel psychologically safe to try new methods; and d) there is sufficient time and space for reflective thinking and evaluation.	A climate in which: a) leaders express their own fallibility and need for team members' assistance and input; b) team members feel

			that they are essential, valued, and knowledgeable partners in the change process; c) individuals feel psychologically safe to try new methods; and d) there is sufficient time and space for reflective thinking and evaluation.
E	Readiness for Implementation	Tangible and immediate indicators of organizational commitment to its decision to implement an intervention.	Overt indication that an organisation is committed to implement genomics in nephrology
1	*Leadership Engagement*	Commitment, involvement, and accountability of leaders and managers with the implementation.	Commitment, involvement, and accountability of leaders and managers with the implementation of genomics in nephrology
2	*Available Resources*	The level of resources dedicated for implementation and on-going operations, including money, training, education, physical space, and time.	Resources dedicated for implementation and on-going operations, including money, training, education, physical space, and time.
3	*Access to Knowledge & Information*	Ease of access to digestible information and knowledge about the intervention and how to incorporate it into work tasks.	Ease of access to digestible information and knowledge about genomics in nephrology

			and how to incorporate it into work tasks.
**IV. CHARACTERISTICS OF INDIVIDUALS**			
A	Knowledge & Beliefs about the Intervention	Individuals' attitudes toward and value placed on the intervention as well as familiarity with facts, truths, and principles related to the intervention.	Individuals' attitudes toward and value placed on the use of genomics in nephrology as well as familiarity with facts, truths, and principles related to the intervention.
B	Self-efficacy	Individual belief in their own capabilities to execute courses of action to achieve implementation goals.	Individual belief in their own capabilities to deliver genomics in nephrology
C	Individual Stage of Change	Characterization of the phase an individual is in, as he or she progresses toward skilled, enthusiastic, and sustained use of the intervention.	The phase of an individual as they progress to becoming skilled and enthusiastic about sustained use of genomics in nephrology
D	Individual Identification with Organization	A broad construct related to how individuals perceive the organization, and their relationship and degree of commitment with that organization.	How the individual perceives the nephrology or genetics team, and the extent of the commitment
E	Other Personal Attributes	A broad construct to include other personal traits such as tolerance of ambiguity, intellectual ability, motivation, values, competence, capacity, and learning style.	Personal traits e.g. tolerance of ambiguity, intellectual ability, motivation, values, competence, capacity, and learning style.

V. PROCESS			
A	Planning	The degree to which a scheme or method of behavior and tasks for implementing an intervention are developed in advance, and the quality of those schemes or methods.	The degree of planning taken place before the implementation of genomics in nephrology and the quality of the planning
B	Engaging	Attracting and involving appropriate individuals in the implementation and use of the intervention through a combined strategy of social marketing, education, role modeling, training, and other similar activities.	The process of engaging others in the implementation of genomics in nephrology, e.g. social marketing, education, role modeling etc
1	*Opinion Leaders*	Individuals in an organization who have formal or informal influence on the attitudes and beliefs of their colleagues with respect to implementing the intervention.	People in the organisation or nephrology or genetics team who have influence over their colleagues relating to the implementation of genomics in nephrology
2	*Formally Appointed Internal Implementation Leaders*	Individuals from within the organization who have been formally appointed with responsibility for implementing an intervention as coordinator, project manager, team leader, or other similar role.	Formally appointed implementation roles e.g. coordinator, project manager, team leader
3	*Champions*	"Individuals who dedicate themselves to supporting, marketing, and 'driving through' an [implementation]" [101] (p. 182), overcoming indifference or resistance that the intervention may provoke in an organization.	"*Individuals who dedicate themselves to supporting, marketing, and 'driving through'* " *Genomics in nephrology*" [101] (p. 182), overcoming

			indifference or resistance that the intervention may provoke in an organization.
4	*External Change Agents*	Individuals who are affiliated with an outside entity who formally influence or facilitate intervention decisions in a desirable direction.	People from outside the organisation or nephrology or genetics care team who formally and positively influence or enable implementation of genomics in nephrology
C	Executing	Carrying out or accomplishing the implementation according to plan.	The process of implementation in line with the plan
D	Reflecting & Evaluating	Quantitative and qualitative feedback about the progress and quality of implementation accompanied with regular personal and team debriefing about progress and experience.	Feedback about how implementation of genomics in nephrology is going including personal and team perspectives

Table S2: Spreadsheet for CFIR Coding analysis

<table>
<tr><th>Barrier</th><th>Quote from text</th><th>CFIR code</th><th>Matched intuitive intervention from the interview transcripts</th><th>Quote from text</th><th>Theory informed intervention (from CFIR-ERIC)</th></tr>
<tr><td>Long wait-time</td><td></td><td></td><td></td><td></td><td></td></tr>
<tr><td>Long waiting time for clinic</td><td>They are very busy. There is often a long waitlist to get seen there.</td><td colspan="3" rowspan="7">Inner setting: available resources
Funding for testing and clinic
Funding support for the test and consultation is also very important.</td><td></td></tr>
<tr><td>Long waiting time for clinic</td><td>The problem is because of the limited funding opportunity, the waiting time for this clinic is over 6 months. It is one of the things that I have been trying to obtain funding for, but it has been extremely difficult.</td><td></td></tr>
<tr><td>Long waiting time for clinic</td><td>When the result comes back, they may or may not have a timely follow-up, probably being their biggest frustration.</td><td></td></tr>
<tr><td>Long waiting time for clinic</td><td>Patients still get seen but [it] takes ages.</td><td></td></tr>
<tr><td>Long waiting time for clinic</td><td>The wait times are long. Clearly most of the work we do is not urgent, so there would be several months as a wait time …</td><td></td></tr>
<tr><td>Long waiting time for clinic</td><td>The other thing in terms of current delays is one has to recognise the interference that COVID had in the whole process with clinics being cancelled or being deferred.</td><td></td></tr>
<tr><td>Long waiting time for clinic</td><td>… my experience is that the waiting list can be quite long and patients have reported back saying there is a 3-6 month wait for an opinion.</td><td></td></tr>
</table>

Long waiting time for clinic	There have been a few where we have linked in with genetics, mainly at XX because we have the counsellors available, although the waiting list can be a bit long.		
Long waiting time for clinic	...their main concern was that [it] takes quite a while to be seen in the clinic.		
Long waiting time for clinic	Obviously there is always a time delay with counselling and so forth.		
Long turn-around time for results	... and then several months' worth delay to get any turn around for the testing which I think is a second issue.	**Intervention characteristics: adaptability**<br>**Funding for testing and clinic**<br>**Funding support for the test and consultation is also very important.**	
Long turn-around time for results	They probably found it a bit frustrating. In that they have the test and then there is a long lag time for the result.		
Long turn-around time for results	... often to get the results back, again they are often sent interstate and sometimes even internationally, so the timing of the feedback is the most outstanding and universal commentary from that point of view.		
Long turn-around time for results	It is not a quick turnaround test and the communication around that probably has need for improvement.		
Long turn-around time for results	I don't know if it has changed but [it] certainly [takes] longer for tests to be done.		

Long turn-around time for results	The other thing is that the tests take a long time to come back, so that’s one common feedback from the patients as well.				
Long turn-around time for results	An ongoing issue but is an issue because these tests get sent elsewhere and therefore the results take time to come back. I think the genetics clinic is quite a niche clinic and people go in there expecting not to know the answer then and there, and when their bloods get sent off, they know it will take many months for the results to come back, so I guess people go in there knowing it but it takes a long time.				
Poor communication about timing of results	Unfortunately nothing really came out of that. I think it is more a patient factor as she lost interest by the time she had to go and get the test and see somebody else in a different part of XX. By that stage she had lost interest in getting it done.	**Outer setting: Patient Needs and resources**			
Poor communication about timing of results	It is not a quick turnaround test and the communication around that probably has need for improvement.				**Involve patients/consumers and family members**
**Costs related to genomics service**					

Lack of funding	You need to demonstrate that it makes a difference to the patient in front of you but it has to be cost-effectiveness but also alters management. When we have patients that are time poor or even out of district for whatever reason, it is really hard to justify that when there is no end point.	**Intervention characteristics: Cost**<br>**Funding for testing and clinic**<br>**Funding support for the test and consultation is also very important.**	
Lack of funding	They have set up a genetics department as you know, and have been supportive of the renal department getting involved in that but they haven't committed huge amounts of money because they don't have huge amount of money.		
Lack of funding	I'd imagine a combination of limited staff, specialised need and funding – the usual issues.		
Lack of funding	It would be nice have some security for overall funding long-term.		
Lack of funding	We have always respected the XX position and the fact they have set up a state-wide service but they probably do need more funding and resources to implement some of their models of care.		
Lack of funding	I would say that we are fairly dysfunctional in the genetics component, largely perhaps due to a combination of personnel, infrastructure and funding being the reasons.		

Lack of funding	Funding is tricky. You probably need to show there is a role in what you are doing and there is a benefit rather than we just test more people but at the end of the day, we don't really know what that's going to show. What comes first, give us money and then we will show you an outcome but we need to see an outcome before you get funding. That makes quite a tricky concept sometimes for a lot of areas of research.		
Lack of funding	We would like it to be more often but the funding for that, we will have to see.		
Lack of funding for testing	… really this is a funding issue. Because you can't get funding for anything except Alport's, so it has [to] go through our genetics clinic, so they might pay for it. That includes all as you know cystic diseases and you can't get any money for it.	**Intervention characteristics: Cost**<br>**Funding for testing**<br>**Funding support to get the test done to be able to pay for the cost of the genomic test which probably needs to happen at the central level. Medicare reimbursement type process but that doesn't seem to exist.**	
Lack of funding for testing	All of these tests are pretty expensive and the Government then will be very reluctant to keep funding things.		
Lack of funding for testing	We also have access to testing at the XX but again there is also funding issues.		

Lack of funding for testing	That has been my only real genomic testing exposure in the polycystic kidney area and with AHUS, that has been something relatively more recent, whereby if one needed to do that testing, it would usually need to go Interstate and there is a current price tag of about AUD15,00. The first question that the institution ask, is where is the money going come from to pay for that and often there is often the justification that needs to be generated locally and it can be approved but there is always those difficulties		
Lack of funding for testing	I think it has minimal funding. We had a talk from XX. Basically there has limited funding, so they have to triage and prioritise investigations, is my understanding.		
Lack of funding for testing	We don't have much funding from the hospital for testing. There is a very small amount of money given to VCG to decide as what should be done or not. They relegate quite a lot of that funding towards cancer genomics and very little spent towards other nephrology work.		
Lack of funding for testing	There is a very small amount of money that the department will allocate to genetic tests.		

Lack of funding for testing	I think it is largely an issue, is my understanding. I think the original set up of the clinic was funded and there were funds for testing but I think this funding is no longer available or much harder to get.		
Lack of funding for clinic	The problem is because of the limited funding opportunity, the waiting time for this clinic is over 6 months. It is one of the things that I have been trying to obtain funding for, but it has been extremely difficult.	**Intervention characteristics: Cost**<br>**Funding for clinic**<br>**Funding support for the test and consultation is also very important.**	
Lack of funding for clinic	If it was up to the nephrology department and funding challenges of the nephrology department and our management group, this clinic would not exist.		
Lack of funding for clinic	It is very clunky the way it is set up. It is not a funded clinic and is not incorporated in usual hospital outpatients and is such a small component.		
Lack of funding for clinic	I think it is largely an issue, is my understanding. I think the original set up of the clinic was funded and there were funds for testing but I think this funding is no longer available or much harder to get.		
Lack of funding for staff	We would love to and there are potential people who are extremely suited for the job but		

	we don’t have enough money to create another position.				
Lack of funding for staff	We do it from our personal interest. We don’t have any nurse support or any other support for this. This is all pure clinical interest on my part of the paediatric nephrologist’s part. So we book the patients ourselves and bring them in. There is no funding.				
**Clinical utility of genomics**					
Perceived impact of results	There is a whole lot of uncertainty – firstly with indication, secondly when they get [it] what test should be ordered, and if they come back what the implications are for the patient, and how much can actually be done about that.	**Characteristics of individual: knowledge and beliefs about the intervention**<br>**Genomics Champion**<br>**The most important ones really are a Genomics Champion, which we have but we really need that pushed along.**			
Perceived impact of results	In most instances I might be able to offer my patient the story that that they have got new or unusual mutation but outside of that, unless there is a clear therapeutic pathway which is available to them, it is not high-value meaningful information, and then it is then competing with information around a whole range of other things.				

Perceived impact of results	...unless we have A, clear therapeutic targets or B, clear prognostic pathways, it is not something you can easily sell to people.		
Perceived impact of results	Whether that is a lack of evidence of whether lack of knowledge of the evidence that's out there. I certainly don't feel it is prominent in our journal clubs or discussion about what certain subgroups of clinical presentations where genetic testing could help with those.		
Perceived impact of results	The initial barrier of just finding out where to send them and what tests to send is compounded by the "what would be do with that anyway afterwards".		
Perceived impact of results	I don't think clinicians are barriers as such but just unsure currently as to the role [of genomic testing] but again my perspective would be very different to a paediatric nephrologist working in the genetics clinic at XX.		
Perceived impact of results	Some of that's probably driven by clinical relevance Vs research interest, if that makes sense.		

Perceived impact of results	Probably if anything, they are very dissatisfied with the outcome, as the patients who have reported back to me, say they have got nothing out of that service or the testing with the education being poor. So I guess I am a little bit jaded by feedback from them and the experience that I have had in regards to the benefit of that clinic and that service.		
Perceived impact of results	There are difficulties in implementation at the moment because it hasn't found its place, I don't think.		
Perceived impact of results	I can't think of where there has really been an outcome where somebody has walked away saying this testing has showed something which is really beneficial and it has changed my clinical management or testing of my family or something like that.		
Perceived impact of results	You probably need to show there is a role in what you are doing and there is a benefit rather than we just test more people but at the end of the day, we don't really know what that's going to show.		

Perceived impact of results	Genetics is always going to be hard to give a patient a definite answer on, but presumably that will improve as we collect more data, so definitely that may be a barrier for wanting testing as well, if they understand that they may not get an answer.				
Perceived impact of results	I guess there is not a lot to offer in terms of therapeutics.				
Perceived impact of results	It was always very tricky to get the students to be interested in genetics, because they found it a bit tricky and also found it difficult to recede relevance.				
Perceived impact of results	...if we had a bit more evidence or idea of what would be best in terms of outcomes for patients. I would be happy with either option depending on which works best. I like interventional nephrology for example, because I know there is evidence that it is just as good as a radiologist				
Perceived impact of results	It is a combination of 'we weren't told much', 'we had some testing done, we didn't get any feedback', or 'we did get feedback and didn't show a thing'.				
**Lack of resources for genomics service**					

Lack of resources	The clinic doesn't stick with us. We don't do the booking, so it is up to the genetics team. They are brilliant but again it is a resource issue, which makes it sometimes difficult in terms of our communication.	**Inner setting: Available resources**<br>**Funding for genomics service**<br>**Funding support – that is going to become critical**	
Lack of resources	The other thing you can do is really re-create an environment where every issue goes across to a genomics or genetics clinic in which case, I think the genomics or genetics capacity is going to be overwhelmed very quickly, because it is – (1) a highly specialised area, (2) a very expensive area, (3) not everyone who has a genetic test is going to benefit from it.		
Lack of resources	I think it would be harder for us to establish it as a management service, because we are probably occupied with the diagnostic arm, to additionally provide long-term care for patients, I think would consume significant resources and detract potentially from our ability to provide the diagnosis.		
lack of resources	There is a clinic but they are very limited in what they can test for, is my understanding.		

lack of resources	We did not have a clinical genetic counsellor until probably about 6 months ago, and one of the genetic counsellors from XX works here part-time (0.5) but then she works here with cancer stuff. She spends some time with us in one clinic but not on a consistent basis, to expect her to work with us is very difficult.		
lack of resources	I'd imagine a combination of limited staff, specialised need and funding – the usual issues.		
lack of resources	We do it from our personal interest. We don't have any nurse support or any other support for this. This is all pure clinical interest on my part of the paediatric nephrologist's part. So we book the patients ourselves and bring them in.		
lack of resources	We have always respected the XX position and the fact they have set up a state-wide service but they probably do need more funding and resources to implement some of their models of care.		
Lack of resources	I think firstly is the infrastructure more than anything.		
lack of resources	...the systems set-up needs to change and hopefully will over time within the process of trying to fix that.		

lack of resources	I would say that we are fairly dysfunctional in the genetics component, largely perhaps due to a combination of personnel, infrastructure and funding being the reasons.		
Lack of resources	I think people like it but there is not the time, resources here or probably the expertise to research it or manage them. It is not specifically encouraged I would say, beyond any other condition.		
Lack of resources	There is often a bit of intellectual interest as well feeling like, it would be good for the patient to have more information, but when the clinical gets really busy, sometimes it might go into the too-hard basket,		
Lack of time for learning	We are excited about what the future hold but we probably don't have the on-hand experience and the real time [and are] cautiously optimistic about what implications are down the track.	**Inner setting: Access to knowledge and information**<br>**Genetics training for nephrology trainees as a rotation**<br>**It does need to incorporated into our basic training – absolutely. Six-month rotation could be an option. It should be part of the 3-year course. You may get a minimum amount of time.**	
Lack of time for learning	It is quite difficult for us with all the other clinical commitments that each of us have, and then on top of that COVID has really made things worse for adult medicine.		

Lack of time for learning	...people are already time-poor, the average age of a nephrology trainee is getting older and you have competing priorities on time, so how are you going to put in a completely new area, superimposed on what is already a very grounded curriculum.				
**Interest in genomics**					
Lack of interest	To be honest, we don't have someone who is really passionate about genomics and polycystic kidney disease as an example.				
Lack of interest	Whereas with the genetics clinic, I feel like there is probably going to be a lot of barriers to setting one up, particularly if there is not a lot of interest from the nephrology department.				
lack of interest	The rotation would only be of interest to some. I don't think everybody would necessarily jump at it.	**Process: champions**<br>**Genomics Champion**<br>**The most important ones really are a Genomics Champion, which we have but we really need that pushed along.**			
Lack of interest	A bit tricky here driven by lack of interest and dysfunctional personalities and the systems set-up needs to change and hopefully will over time within the process of trying to fix that.				
lack of interest	It was always very tricky to get the students to be interested in genetics, because they found it a bit tricky and also found it difficult to recede relevance.				

**Genomics knowledge amongst clinicians**					
lack of theoretical knowledge	Whether that is a lack of evidence of whether lack of knowledge of the evidence that's out there. I certainly don't feel it is prominent in our journal clubs or discussion about what certain subgroups of clinical presentations where how genetic testing could help with those.				
lack of theoretical knowledge	We are starting from nothing really. There is nothing currently at the moment. Really it is such a low position, that what you need now is a greater appreciation of its importance and offer uptake course and kidney schools so people are used to it.	**Inner setting: Access to knowledge and information**<br>**Educational meetings**<br>**Some incorporation of more education. Update course and kidney school**			
lack of theoretical knowledge	I think that needs a lot more discussion and a lot more presence at national meetings and ANZSN. It is something that came to a lot of specialities recently, and nephrology, particularly in Australia hasn't done a lot with it.				

lack of theoretical knowledge	We can talk about interventions but before we start identifying barriers, to my mind, the major interventions here would be a lack of awareness really, in terms of what genetics and genomics can offer presently, and what it is likely to offer in the future.	**Inner setting: Access to Knowledge and Information**<br>**Provide training**<br>**There probably needs to be some sort of training so you are competent. It is a lot more than just interpreting. It is not just knowing what to do with a sequence result that comes back to you. We have to be involved in counselling of people which is a huge area. Really, it sort of has us linking in with another service which I think has totally worked out to medical genetics really. I think there probably has been some sort of a training pathway or some sort of thing that shows you are competent in all areas of medical genetics.**	
lack of theoretical knowledge	In terms of the genetics clinic, I don't know the outcomes. Are renal genetics just nephrologists with an interest, is that good enough for a patient or do they deserve better with the outcomes better with a true geneticist, I don't really know the answer.		
lack of theoretical knowledge	I think the genetics clinician is probably better in also counselling patients. I am not sure if nephrologists will have all the adequate training or experience to do all the counselling etc.		

lack of theoretical knowledge	I think it is something that I am interested in but is something that we [don't] get that much exposure as trainees given our curriculum that the College sets. There is already so much with dialysis, transplantation and I think it is always hard to learn genomics as an added-odd topic.		
lack of theoretical knowledge	We are excited about what the future hold but we probably don’t have the on-hand experience and the real time cautiously optimistic about what implications are down the track.		
lack of theoretical knowledge	I don’t think there are too many nephrologists that have great expertise in genetics and genomics, so that is a problem except for a few like me who are interested. It is a very new domain as well.		
lack of theoretical knowledge	That is a common opinion across domains that people do not know how to do or understands things.		
lack of theoretical knowledge	The knowledge amongst the new era trainees in genomics and genetics, is (I won’t say poor) but I don’t think they have adequate exposure.		
lack of theoretical knowledge	Basic genetic information of the clinicians I think is the key factor.		
lack of theoretical knowledge	I speak to most trainees who say we don’t have a lot of familiarity with renal genetics.		

lack of theoretical knowledge	There is a lot of content and it becomes quite subspecialised when you start going into genetics and all of that.		
lack of theoretical knowledge	My impression is that when someone walks in without significant background knowledge, it remains just a curiosity but without much appreciation.		
lack of theoretical knowledge	The barrier would be my lack of knowledge.		
lack of theoretical knowledge	So it comes back to our understanding and knowledge in all of those areas.		
Lack of knowledge about process	At the moment we are pretty generic in the way we have advertised – just refer anyone that you are not sure of the cause of their kidney failure.	**Inner setting: access to knowledge and information**<br>**Develop educational materials**<br>**Have a clear form, what you need to do, what we can offer, cost, approximate waiting time and [if] the result will be discussed by Geneticists and Counsellor or both.**	
Lack of knowledge about process	I think the risk you have right now is that, if you open up these barriers without actually improving in the knowledge in the group which is going to be ordering these tests, then you are likely to get a lot of noise and a lot of excess testing or requests for testing which overwhelms your Units without actually achieving good outcomes for the patients.		

Lack of knowledge about process	There is a renal genetic service at XX where they have a clinic, so I have referred a couple of patients there from time to time. As far as my awareness of what they actually do, it is very little. Don't know what they actually do. Other than seeing the patient with a possible inherited disease, I don't know how they investigate them all.		
Lack of knowledge about process	I think it is matter of people having the foresight and vision to actually to do that. Most of these patients fall through the cracks. The clinics are there, may be need to be advertised or reemphasized in some way in our department.		
Lack of knowledge about process	I know some of them go to the Children's Hospital at XX and I have got their form. Beyond that I don't really know what I am supposed to do with these people.		
Lack of knowledge about process	There is always an issue I guess, gaining attraction in regional areas and private practice. This is the case for genomics but also in transplantation, where because people aren't regularly exposed to discussions that refer to the availability or utility of prospect of genetic testing, that they are less likely to refer.		

Lack of knowledge about process	There are a few people that have an interest which is really helpful but there are a lot who – the initial barrier of just finding out where to send them and what tests to send is compounded by the "what would be do with that anyway afterwards".		
Lack of knowledge about process	In general, it is a little bit of an ill-defined area. Both in terms of access to the service but also knowing who we should refer to.		
Lack of knowledge about process	I think executive summary, very early on we all think it is going to be important in the not too distant future but currently most people are unsure what tests are out there, who we should be referring to, and exactly how we should doing it – in terms of the logistics of doing including right paperwork or the right person.		
Lack of knowledge about process	Everyone is aware of the clinic but I don't know if they actively refer to the clinic because of the fact that it is not quite so visible.		
Lack of knowledge about process	The barriers to renal genetics testing in the General Clinic is that when we would refer, that clinic may say that we don't think that this qualifies for genetic sequencing. There were one or two individuals that were affected where cases were well defined, where we think there might be something generic and I		

	would say no… so one of the barriers is seems a little bit arbitrary at least in the Generic Clinic as to what gets accepted and what doesn’t.				
Lack of knowledge about process	The issues are around the clinicians being aware of which patients would benefit from going to the clinic and then remembering that information when they are seeing the patients.				
Lack of knowledge about process	It is a matter of us knowing when we should refer and doing that at a timely fashion.				
Lack of knowledge about process	There is a whole lot of uncertainty – firstly with indication, secondly when they get it what test should be ordered.				
**Genetics expertise**					
Lack of genetics expertise	It is state-wide service, so we refer to the XXX so therefore we don’t really have anyone in house that’s a renal genetics person so that might limit our ability to be involved in trials and things like that pertaining to that.	**Inner setting: Available resources**<br>**Easier access to local genetics expertise**<br>**Easy access locally. It would be really good even if we had a genetics counsellor locally, so once we get some results back to talk through what they mean with the patients, and also help us interpret what the test is might be really useful**			

Lack of genetics expertise	I suppose that's one my issues in terms of with any sort of genetic testing we do, my personal fear is that it should go through such a multidisciplinary genetic clinic so that the patient can be appropriately counselled as to the implications of the results		
Lack of genetics expertise	This clinic I am referring to is in another Unit in a different local health district. The EMR systems for example are not linked well, so there is some trouble getting access to past pathology results and clinical documentation and so forth. If we could keep things local, that would be much easier.		
Lack of genetics expertise	I think people like it but there is not the time, resources here or probably the expertise to research it or manage them.		
Lack of genetics expertise	Unfortunately nothing really came out of that. I think it is more a patient factor as she lost interest by the time she had to go and get the test and see somebody else in a different part of XX. By that stage she had lost interest in getting it done.		
Lack of genetics expertise	Even if there is funding, it is very hard to recruit people to work in XX and I don't think any clinical geneticist is ever going to set foot in XX to work.		

Lack of genetics expertise	It may be the preferred Model, because we don't know if we have enough Clinical Geneticists to be able to refer every single patient to.				
**Role for genomics service**					
Perception of need for service	The number of genetic problems we would get to see is not very big, so I would say that we probably will have about 10 referrals in a year and a whole lot of that is actually paediatric, but we work only 0.5 with the paediatric department, so that means that is going to be quite a bit of trouble for her to book clinics.				
Perception of need for service	I don't think there is enough business and scope to have a whole single nephrology genetics clinic, certainly on a weekly basis or perhaps even a monthly basis.				
Perception of need for service	As currently configured, there is not enough work to support someone just doing this for 6 months or a year. I can't see what they would do much of the time.	**Inner setting: Tension for change**			
Perception of need for service	The worry for me in setting up sub-specialties is that people develop a silo mentality and they off and say to the rest of the people in nephrology "you are too stupid to understand what we are doing here".				**Conduct local needs assessment/facilitate relay of clinical data to providers**
